# Supplementary material for: Collaborative intelligence in AI: Evaluating the performance of a council of AIs on the USMLE
Source: PLOS Digit Health. 2025 Oct 9;4(10):e0000787. doi: 10.1371/journal.pdig.0000787 (PMC12510544; doi:10.1371/journal.pdig.0000787)
Supplement: S2 File — (ZIP) [file pdig.0000787.s002.zip › S2 Data Raw data files containing unprocessed question inputs and LLM outputs including transcripts of deliberations/STEP3_Question-72-1_Type-MC-NJ.docx]

**Facilitator** **[Round 1]:** A 35-year-old man was admitted to the hospital 3 days ago because of pneumonia, but he has shown no clinical improvement despite ceftriaxone and azithromycin therapy. Upon admission, the patient exhibited blood-streaked sputum and chest x-ray showed hilar adenopathy. Medical history is otherwise unremarkable. The patient is a construction worker and he returned home 1 week ago from the southwest United States, where he had been temporarily employed. He does not smoke cigarettes. BMI is 20 kg/m². Vital signs today are temperature 38.5°C (101.3°F), pulse 106/min, respirations 22/min, and blood pressure 110/70 mm Hg. Pulse oximetry on room air shows an oxygen saturation of 95%. PPD skin test is nonreactive. Acid-fast smears of three sputum samples were all negative. Complete blood count at the time of admission showed a normal leukocyte count with 15% eosinophils. Results of blood culture have been negative since admission. Which of the following is most likely to provide a diagnosis? (A) Bacterial culture of sputum (B) CT scan of the chest (C) Fungal culture of the blood (D) Serologic study for Coccidioides immitis (E) Sputum cytologic study (F) Urine assay for Legionella antigen

**AI_A:** (1) RESTATING THE QUESTION: The patient is a 35-year-old man with blood-streaked sputum and pneumonia that is not responding to conventional antibiotic treatment. We're trying to identify the best next step to establish a diagnosis, considering the patient's recent travel history, occupational exposure, and clinical presentation. (2) PLANNING: The assumptions are that our patient has a bacterium that is resistant to azithromycin and ceftriaxone or a different type of pathogen altogether. Further assumptions include the potential relevance of the specifics related to the patient's occupation, geography, or unknown etiological agents. An alternate assumption might be that this disease process is the result of fungal infection, given his occupational exposure as a construction worker and recent trip to the southwest United States. (3) CLINICAL REASONING: His pneumonia hasn't responded to the ceftriaxone and azithromycin, so these treatments may not be effective against the causative pathogen. His elevated eosinophil count might suggest an allergic reaction or parasitic infection, but the fact that he doesn't smoke makes occupational lung disease a prioritized consideration. Moreover, his recent return from the southwest United States may suggest a potential exposure to a geographically specific pathogen like Coccidioides immitis, which is endemic to that region. Coccidioidomycosis could explain his pneumonia, increase in eosinophils, resistance to antibiotic therapy, and failure to improve despite treatment. (4) CONCLUDING: Connecting these clinical features and the epidemiologic clues from his travel and occupational history, it is plausible to consider a fungal etiology for this patient's pneumonia, specifically Coccidioides immitis. (5) RESPONSE: Therefore, the most likely diagnosis might be established through a (D) Serologic study for Coccidioides immitis, which would unequivocally verify the etiology of this atypical pneumonia and guide us to specifically appropriate antifungal treatment.

**AI_E:** RESTATING THE QUESTION: We're looking at a 35-year-old male construction worker who was admitted to the hospital with pneumonia that hasn’t shown improvement despite antibiotic treatment. Some notable medical findings include blood-streaked sputum, hilar adenopathy seen in chest X-ray, eosinophilia, and recent travel history to the southwest United States. His bacterial cultures and acid-fast smears have all come back negative. We're now seeking the next most feasible diagnostic test that can help ascertain the cause of his persistent pneumonia. PLANNING: Attendance to his clinical history and presentation, including his occupation, travel history, and symptoms, leads us to question the current diagnosis and treatment for bacterial pneumonia. Apparently, they're unproductive. His profession of a construction worker, recent travel to the southwest United States, and persistent pneumonia could suggest an inhalational fungal pathology, such as Coccidioidomycosis (Valley Fever). The presence of eosinophilia in the blood could be another pointer towards a fungal infection. CLINICAL REASONING: Coccidioides species of fungi are endemic to the desert regions of the southwestern US and often cause pneumonia when their arthroconidial spores are inhaled from disturbed soil, an exposure possibly linked with his construction work. Chest X-ray findings, including hilar adenopathy, are non-specific and might suggest this fungal infection — a diagnosis that aligns with his occupational exposure and travel history. The routine pneumonia treatment with antibiotics such as Ceftriaxone and Azithromycin would not be effective against Coccidioides organisms, hence the lack of clinical improvement. CONCLUDING: Considering these, I hypothesize that a serologic study for Coccidioides immitis can be more helpful than the provided alternatives. A positive test could confirm infection with these specific fungi and could explain the patient's persistent symptoms despite antibiotic treatment for bacterial pneumonia. RESPONSE: Hence, the test most likely to provide a diagnosis in this specific case would be (D) a serologic study for Coccidioides immitis.

**AI_D:** 1) RESTATING THE QUESTION: The case is about a 35-year-old male patient, employed in construction, who was admitted to the hospital for pneumonia. Despite antibiotic therapy with ceftriaxone and azithromycin, his condition did not improve. He has been displaying symptoms such as blood-streaked sputum and his radiological findings reveal hilar adenopathy. He recently returned from southwestern United States. His tuberculosis skin test and cultures for bacteria in the blood have been negative, while his leukocyte count was normal with an eosinophil count of 15%. The question asks which the next most appropriate diagnostic test would be for this patient who is resistant to common antibiotic therapy for pneumonia. 2) PLANNING: The key assumptions to consider are that the treatment has failed because it was ineffective against the causative agent. It is also assumed that the patient has contracted some pathogen in the southwestern United States which may be causing the pneumonia. In this context, the prominence of eosinophils in his blood count might indicate an allergic reaction or a parasitic infection. Among the alternatives, it is necessary to prioritize, based on the most likely organisms and their endemic regions, the need of specific diagnostic tests for those organisms and resistance to common antibiotics. 3) CLINICAL REASONING: The patient's antibiotic therapy was ineffective, indicating that the causative agent might be non-bacterial or resistant to the antibiotics used. The patient's history of travel to the southwestern United States points toward possible exposure to certain pathogens common in that region, such as Coccidioides immitis, a fungus known to cause pneumonia-like symptoms and be resistant to common antibiotics. The notable eosinophilia could be due to a parasitic or fungal infection. Acid-fast bacillus smear, and culture were negative, excluding tuberculosis and typical bacteria. 4) CONCLUDING: Considering the patient's recent travel history, continued symptoms despite broad-spectrum antibiotics, eosinophilia, and hilar adenopathy, an infectious process caused by an organism endemic to the southwestern United States seems most probable. Among the tests listed, a serologic study for Coccidioides immitis would be the most useful in providing a diagnosis, as it is endemic to that region and causes symptoms similar to the patient's. 5) RESPONSE: Given the patient's non-improvement on antibiotic therapy, eosinophilia, and travel history to southwestern United States, the most likely test to help provide a diagnosis is (D) Serologic study for Coccidioides immitis.

**AI_B:** RESTATING THE QUESTION: The primary concern is about a 35-year-old man who is suffering from pneumonia. Despite administering empirical antibiotic treatments for pneumonia such as ceftriaxone and azithromycin, there is no significant improvement in his clinical condition. Some characteristics of his condition include bloody sputum and hilar adenopathy (which is a swelling or enlargement of the lymph nodes in the lungs), as identified during a chest X-ray examination. Acid-fast smears, a test for specific bacterial infections like tuberculosis, have tested negative. His leukocyte count is within the normal range, but he has an increased level of eosinophils (a type of white blood cells usually increased in an allergic reaction or parasitic infection). He recently returned from a trip to the southwest region of the United States. The question is now about which diagnostic method would be most useful to identify the culprit in his condition among the provided choices. PLANNING: The lack of response to antibiotic therapy suggests a pathogen not susceptible to this treatment, for example a fungal or viral infection, or a bacterium not covered by these antibiotics. The patient's high eosinophil count could also suggest a fungal infection as they are often increased in response to fungal pathogens. His recent travel history to the southwestern United States and occupation as a construction worker further supports a fungal infection due to environmental exposures like dust and soil, which are reservoirs for several types of fungi, including Coccidioides species. Thus, the clinical priority should focus on identifying a likely fungal pathogen. CLINICAL REASONING: Based on the patient's lack of response to empirical antibiotics, one might question the presence of bacterial pneumonia. Given the normal leukocyte count but high eosinophil percentage, along with the background of travel to the southwestern United States and his profession as a construction worker, points towards the possibility of fungal etiology. Possible fungal pathogens linked to this region and occupational exposure can include Coccidioides species, which usually cause pulmonary infections that can present similar to pneumonia. Thus, connecting these dots, an evaluation for a possible fungal infection should be pursued. CONCLUDING: The more likely reason for the patient's condition remains a fungal lung infection despite the negative acid-fast smears and bacterial blood cultures. Coccidioidomycosis, which is endemic to the southwestern United States and related to occupational exposure in dust and soil, could be a plausible cause. Selection of a test that can ascertain the presence of a fungal agent, specifically Coccidioides, among the provided choices should be considered. RESPONSE: The appropriate choice to facilitate diagnosis in this case would be (D) Serologic study for Coccidioides immitis. This study will help in detecting antibodies to coccidioidal antigens, clarifying the presence of Coccidioides species, hence leading to the right diagnosis.

**AI_C:** RESTATING THE QUESTION: This is a case of a 35-year-old healthy man who works in construction and was recently working in the southwest United States. Despite being on pneumonia-targeted antibiotics, his pneumonia-like symptoms haven't resolved.The patient has fever, tachycardia, and high percent of eosinophils in the blood. Sputum samples have returned negative for bacterial cultures, and the PPD skin test is nonreactive. Also, chest x-ray shows hilar adenopathy. The question is asking which of the following diagnostic methods is most likely to reveal the cause of these symptoms? PLANNING: I'll first explain each of the diagnostic methods options, then plan how to approach answering the question. (A) Bacterial culture of sputum would identify bacterial pneumonia, but since the patient hasn't improved on current antibiotics, and three sputum samples have been negative for bacteria, bacterial infection is unlikely. (B) A CT scan of the chest could provide more detailed image than an x-ray, potentially revealing masses or nodules. (C) Fungal culture of the blood can diagnose systemic fungal infection, which could be considered given the patient's work history and location. (D) Serologic study for Coccidioides immitis can provide evidence of Coccidiomycosis, a fungal infection common in certain parts of the US. (E) Sputum cytologic study can look for cancerous cells or signs of certain lung diseases. (F) Urine assay for Legionella antigen can diagnose Legionnaires' disease, a type of pneumonia. CLINICAL REASONING: Given the patient's job, travel history and the presence of eosinophilia in the blood, Coccidioides immitis infection likely should be suspected. Furthermore, since the patient hasn't responded to antibiotics and bacterial cultures and acid-fast smears from his sputum samples have been negative, bacterial and mycobacterial infections are unlikely. Therefore, the CT scan and bacterial, mycobacterial and Legionella tests probably won't provide a definitive diagnosis. Since there's no evidence suggesting cancer, a cytologic study also likely won't be particularly revealing. CONCLUDING: The current clinical picture suggests a fungal infection more than any other pathologies. The patient's response to treatment, travel history, and eosinophilia in the blood, all add points towards this direction. RESPONSE: Hence, the most suitable test in this context is a Serologic study for Coccidioides immitis (D), which is endemic to the Southwestern United States and can cause pneumonia-like symptoms, especially in individuals who work in soil-disturbing activities such as construction.

**Facilitator** The consensus among the AI responses is that the most appropriate next step in diagnosing the 35-year-old construction worker's persistent pneumonia, which has not responded to conventional antibiotic treatment, is a serologic study for Coccidioides immitis (Option D). This conclusion is based on several factors. Firstly, the patient's lack of response to antibiotics suggests that the causative pathogen may be resistant to these treatments or may not be bacterial in nature. Secondly, the patient's elevated eosinophil count could indicate an allergic reaction or a parasitic or fungal infection. Thirdly, the patient's recent travel to the southwest United States and his occupation as a construction worker, which involves exposure to dust and soil, increase the likelihood of a fungal infection, specifically Coccidioidomycosis. This fungal infection is endemic to the southwestern United States and can cause pneumonia-like symptoms. Therefore, given the patient's clinical presentation, occupational exposure, and travel history, a serologic study for Coccidioides immitis is the most likely to provide a definitive diagnosis and guide appropriate antifungal treatment.
